# Mitochondrial Genomes Provide New Phylogenetic and Evolutionary Insights into Psilidae (Diptera: Brachycera)

**DOI:** 10.3390/insects13060518

**Published:** 2022-06-01

**Authors:** Jiale Zhou, Ding Yang

**Affiliations:** Department of Entomology, College of Plant Protection, China Agricultural University, Yuanmingyuan West Road, Beijing 100193, China; zhou_jl1994@163.com

**Keywords:** rust flies, Chylizinae, Psilinae, mitochondrial genome, phylogeny

## Abstract

**Simple Summary:**

Members of Psilidae are commonly known as rust flies. They constitute the largest family of Diopsoidea, with about 340 species being known worldwide. Several species of Psilidae show agricultural significance due to their severe damage of root crops. However, the systematic relationships and intrafamilial classification of Psilidae remained controversial. To provide further information on the phylogeny and evolution of Psilidae, mitogenomes of 6 psilid species are sequenced. Comparative analyses of the 6 newly obtained mitogenomes are conducted. Phylogenetic analyses based on the 6 psilid mitogenomes and public data are carried out, resulting in a monophyletic Psilidae and a non-monophyletic Diopsoidea. The sister relationship between Psilinae and Chylizinae is highly supported. This study provides several new insights into the phylogeny and evolution of Psilidae.

**Abstract:**

Psilidae (Diptera: Brachycera) is a moderate-sized family currently placed in the superfamily Diopsoidea and contains some destructive agricultural and forestry pests. The systematic position and intrafamilial classification of rust flies are in need of further study, and the available molecular data of Psilidae are still limited. In this study, we present the mitochondrial genomes of 6 Psilidae species (*Chamaepsila* *testudinaria* Wang and Yang, *Chyliza bambusae* Wang and Yang, *Chy. chikuni* Wang, *Loxocera lunata* Wang and Yang, *L. planivena* Wang and Yang and *L. sinica* Wang and Yang). Comparative analyses show a conserved genome structure, in terms of gene composition and arrangement, and a highly Adenine plus Thymine biased nucleotide composition of the 6 psilid mitogenomes. Mitochondrial evolutionary rates vary among the 6 species, with species of Chylizinae exhibiting a slower average rate than species of Psilinae. The length, the nucleotide composition, and the copy number of repeat units of the control region are variable among the 6 species, which may offer useful information for phylogenetic and evolutionary studies of Psilidae. Phylogenetic analyses based on 4 mitogenomic datasets (AA, PCG, PCG12RNA, and PCGRNA) support the monophyly of Psilidae, and the sister relationship between Chylizinae and Psilinae, while Diopsoidea is suggested to be non-monophyletic. Our study enlightens the future application of mitogenomic data in the phylogenetic and evolutionary studies of Psilidae, based on denser taxon sampling.

## 1. Introduction

Mitochondria are organelles which play a central role in eukaryotic cell metabolism [1] and bear their own genome (known as mitogenome) [2,3,4]. The mitogenome has become a powerful molecular marker for taxonomic [5,6], phylogenomic [7,8,9,10], phylogeography [11,12], and molecular evolutionary [13,14] studies due to its small size, high copy numbers, relatively simple structure, and rapid evolutionary rate [2,15]. Recent advances in high-throughput sequencing technologies have made it possible to sequence the mitogenome efficiently and cost-effectively [16,17]. Insect mitogenome is a 15 to 18 kb duplex circular DNA, generally encompassing 37 genes (13 protein-coding genes (PCGs), 22 transfer RNA genes (tRNAs), and 2 ribosomal RNA genes (rRNAs)), a control region (CR, or A + T rich region) and several shorter non-coding regions (NCRs) [18]. At present, the mitogenomic data have been extensively used in comparative genomics, phylogenetic and evolutionary analyses of different insect groups, including Diptera [19,20,21,22,23]. Acalyptratae is one of the most diverse lineages of Diptera comprising many species with economic and scientific importance [24,25]. The public mitogenomic data of Acalyptratae, however, mainly focus on Drosophilidae and Tephritidae, while the information about other acalyptrate families is still very limited, which largely impeded our understanding of the phylogeny and evolution of acalyptrate flies.

Psilidae, commonly known as rust flies, is a group of small to medium-sized, yellow or black acalyptrate flies with reduced body setation [26]. Psilids are of economic importance because their phytophagous larvae burrow in the roots, stems, and tubers of plants [26,27,28] and sometimes cause considerable damage on bamboos [29,30], carrots, [31,32] and other root crops [33,34]. Some species have also been reported to induce galls [35,36]. With about 340 species being described so far, Psilidae is distributed in all zoogeographic realms with the highest diversity in the Old World and the Nearctic region, and a few species also occur in the Neotropical region [28,37]. Members of Psilidae are currently assigned into 3 subfamilies (Belobackenbardiinae, Chylizinae, and Psilinae), whereas the genus-level classification within Psilidae has been debated for a long time, especially the status of some generic taxa of Psilinae needs to be reconsidered [27,28,38,39]. In addition, the taxonomic, phylogenetic, and evolutionary studies of Psilidae have largely relied on morphological characters from adults, larvae, and eggs [38,39,40,41], and the comparative and phylogenetic analyses of this family based on molecular data remain unconducted.

The present study offers the mitogenomic data of 6 species of Psilidae, including the first 2 mitogenomes for the subfamily Chylizinae (*Chyliza bambusae* Wang and Yang and *Chy. chikuni* Wang), the mitogenomes of 3 species of the genus *Loxocera* (*L. lunata* Wang and Yang, *L. planivena* Wang and Yang and *L. sinica* Wang and Yang), and that of a species of the genus *Chamaepsila* (*Cha. testudinaria* Wang and Yang). Some of these 6 sampled species, such as *Chy. bambusae*, have been recorded as destructive pests of bamboos [30]. Comparative analysis of the genomic structure, nucleotide composition, substitutional and evolutionary rates among the 6 psilid mitogenomes as well as a molecular phylogenetic study of Psilidae are conducted. This study aims to contribute to our knowledge of the diversity of mitogenome and the phylogeny of Psilidae.

## 2. Materials and Methods

### 2.1. Taxon Sampling and DNA Extraction

Adult flies were collected using swept net in the field and preserved in 100% ethanol at –20 °C before DNA extraction. Detailed collection data were provided in Table S1. Specimens were identified mainly based on the keys, descriptions, and illustrations in Wang [42] and Wang and Yang [43]. Genomic DNA was extracted from thoracic muscle tissues using DNeasy Blood and Tissue kit (Qiagen, Hilden, Germany). The remaining body parts of the sampled specimens were saved as vouchers and deposited in the Entomological Museum of China Agricultural University, Beijing, China. Specimen voucher numbers are included in Table S1.

### 2.2. Mitochondrial Genome Sequencing and Assembly

An Illumina TruSeq library was prepared with 350 bp average insert size and sequenced on the Illumina NovaSeq 6000 platform with 150 bp paired-end reads. The raw reads were trimmed of adapters using Trimmomatic [44], and low-quality and short reads were removed using Prinseq [45]. De novo assemblies of high-quality reads were conducted using IDBA-UD [46], with similarity threshold 98%, and minimum and maximum *k* values of 41 and 141 bp, respectively. Fragments of *COI* near the 5’-terminus (~610 bp) were amplified for each species by polymerase chain reaction (PCR) with primers LCO1490 (5′-GGTCAACAAATCATAAAGATATTGG-3′ forward) and HCO2198 (5′-TAAACTTCAGGGTGACCAAAAAATCA-3′ reverse) [47], and obtained by Sanger sequencing. The *COI* fragments served as bait references to identify the best-fit mitochondrial contigs under BLAST searches [48] with minimum similarity 98%. For checking the assembly accuracy, clean reads were mapped onto the obtained mitochondrial contigs using Geneious 10.1.3 [49].

### 2.3. Mitochondrial Genome Annotation and Analysis

Gene sequences were initially annotated with MitoZ [50], and further corrected in Geneious 10.1.3 [49]. PCGs and rRNA genes were annotated by aligning their sequences with those of homologous genes of other reported Acalyptratae species. The locations and secondary structures of tRNA genes were identified using tRNAscan-SE Search Server (http://lowelab.ucsc.edu/tRNAscan-SE/, accessed on 14 March 2022) [51,52] and ARWEN version 1.2 (http://130.235.244.92/ARWEN/, accessed on 14 March 2022) [53]. Nucleotide composition of mitogenomes and codon usage of PCGs were analyzed with MEGA 7.0 [54]. AT-skew [(A − T)/(A + T)] and GC-skew [(G − C)/(G + C)] were used to measure the nucleotide compositional differences between genes [55]. DnaSP 5.0 [56] was used to calculate the synonymous (Ks) and non-synonymous (Ka) substitution rates of PCGs. Evolutionary rate of PCGs (Ka/Ks, ω) [57,58] was calculated manually.

### 2.4. Phylogenetic Analysis

Including the 6 newly sequenced mitogenomes of Psilidae, a total of 19 acalyptrate mitogenomes were used for phylogenetic analysis (Table 1). Mitogenomes of 2 Calyptratae species were used as outgroups.

The 13 PCGs of each species were aligned separately under the MAFFT algorithm [66] on TranslatorX online platform [67] with the L-INS-I strategy and default setting. Sequence of the 2 rRNA genes was aligned using the MAFFT version 7 online server [68] with G-INS-I strategy. All alignments were verified and checked manually in MEGA 7.0 [54]. Four datasets were prepared for phylogenetic analyses: (1) AA matrix, including amino acid sequences of 13 PCGs (3676 amino acids); (2) PCG matrix, including all 3 codon positions of 13 PCGs (11,028 bp); (3) PCGRNA matrix, including nucleotides in all 3 codon positions of 13 PCGs, and 2 rRNA genes (13,082 bp); and (4) PCG12RNA matrix, including nucleotides in the first and second codon positions of 13 PCGs, and 2 rRNA genes (9406 bp). Heterogeneity of sequence divergence within the 4 datasets was analyzed using AliGROOVE [69] with the default sliding window size.

Phylogenetic trees inferred from the 4 datasets were constructed under Bayesian inference (BI) and maximum likelihood (ML) methods. The site-heterogeneous mixture CAT + GTR model was used for all datasets. BI analyses were performed using PhyloBayes MPI v.1.5a [70]; 2 independent Markov Chain Monte Carlo (MCMC) chains were run after the removal of constant sites from the alignment and were stopped after the 2 runs had satisfactorily converged (maxdiff < 0.3); a consensus tree was computed from the remaining trees combined from 2 runs after the initial 25% trees of each run were discarded as burn-in. ML analyses were performed using IQ-TREE web server (http://iqtree.cibiv.univie.ac.at/ accessed on 14 March 2022) [71] with 1000 bootstrap replicates and automatic model prediction.

## 3. Results and Discussion

### 3.1. General Structure and Nucleotide Composition of Psilidae Mitogenomes

**Figure 1:**
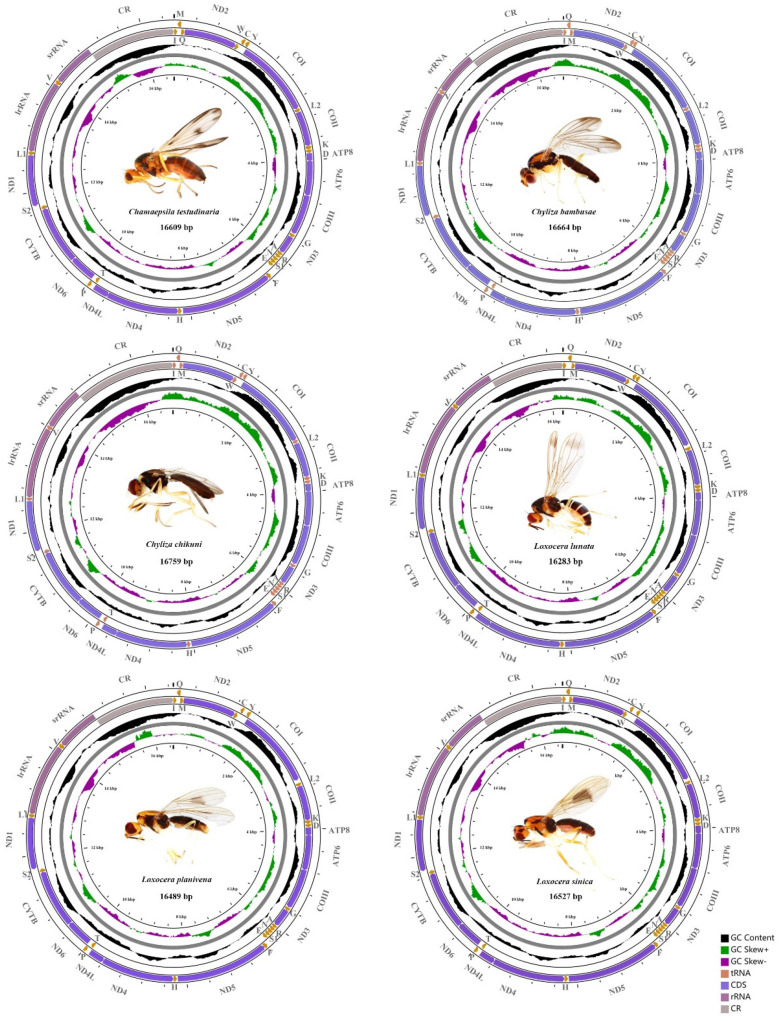
Mitochondrial genomes of *Chamaepsila testudinaria*, *Chyliza bambusae*, *Chyliza chikuni*, *Loxocera lunata*, *Loxocera planivena,* and *Loxocera sinica*. The direction of gene transcription is indicated by the arrows on the strands. Transfer RNA genes are represented by the single letter IUPAC-IUB abbreviations for their corresponding amino acid. Abbreviations: ATP6 and ATP8 for adenosine triphosphate (ATP) synthase subunits 6 and 8; COI–COIII for cytochrome C oxidase subunits I–III; CYTB for cytochrome b; ND1–ND6 and ND4L for nicotinamide adenine dinucleotide hydrogen (NADH) dehydrogenase subunits 1–6 and 4 L; lrRNA and srRNA for large and small rRNA subunits; and CR for control region.

The complete mitogenomes of *Cha. testudinaria*, *Chy. bambusae*, *Chy. chikuni*, *L. lunata*, *L. planivena,* and *L. sinica* are 16,609, 16,664, 16,759, 16,283, 16,489, and 16,527 bp in length, respectively (Figure 1; Table S2). Length differences of the 6 mitogenomes are mainly due to the variable size of the control region. They are compact circular molecules, each containing 37 typical mitochondrial genes (13 PCGs, 22 tRNAs, and 2 rRNAs) and 1 control region. Among these genes, 4 PCGs (*ND1*, *ND4*, *ND4L,* and *ND5*), 8 tRNAs (*trnC*, *trnF*, *trnH*, *trnL1*, *trnP*, *trnQ*, *trnV*, and *trnY*) and 2 rRNAs (*lrRNA* and *srRNA*) are encoded on the minority strand (N strand), while the other 23 genes are located on the majority strand (J strand). The gene order and orientation of the 6 mitogenomes are identical to the typical insect mitogenomes [2,18]. Although mitochondrial gene rearrangements have been reported in several orders of Insecta [18,72,73,74], these events are rather rarely documented in Diptera, which have only been discovered in the mosquitos (Culicidae) [75] and the gall midges (Cecidomyiidae) [76]. Therefore, the mitogenomes of rust flies appear to be conserved and to retain the putative ancestral arrangements [18,21].

The nucleotide composition of the 6 Psilidae mitogenomes (Table 2) is similar, with a high Adenine plus Thymine (A + T) bias (77–80%), which is a common feature of insect mitogenomes [18,77]. The control region has the highest A + T content, while the first and second codon positions of PCGs have the lowest A + T content. Several hypotheses have been proposed to explain the A + T-biased composition heterogeneity [78,79,80], among them the energy efficiency trade-offs [79] is the one tested experimentally. This hypothesis suggests that the synthesis of A and T consumes lesser energy and nitrogen than that of Cytosine (C) and Guanine (G) [80]. All the 6 Psilidae mitogenomes exhibit positive AT-skew and negative GC-skew; the AT-skew ranges from 0.023 (*L. lunata*) to 0.062 (*Chy. bambusae*); the GC-skew ranges from −0.222 (*Chy. chikuni*) to −0.147 (*Cha. testudinaria*). The skewed strand composition is caused by multiple factors, including mutations and selection pressures [21], and the GC-skew value in insect mitogenomes appears to correlate with replication direction [80].

### 3.2. Protein-Coding Genes, Codon Usage, and Evolutionary Rates

Total sizes of the 13 PCGs of *Cha. testudinaria*, *Chy. bambusae*, *Chy. chikuni*, *L. lunata*, *L. planivena,* and *L. sinica* are 11,184 bp, 11,182 bp, 11,182 bp, 11,196 bp, 11,183 bp, and 11,183 bp long, respectively. Each of the 6 mitogenomes exhibit a negative AT-skew of PCGs, ranging from −0.172 (*Chy. bambusae*) to −0.132 (*L. lunata*), and a positive GC-skew of PCGs, ranging from 0.009 (*L. planivena*) to 0.041 (*Chy. bambusae*) (Table 2).

**Figure 2:**
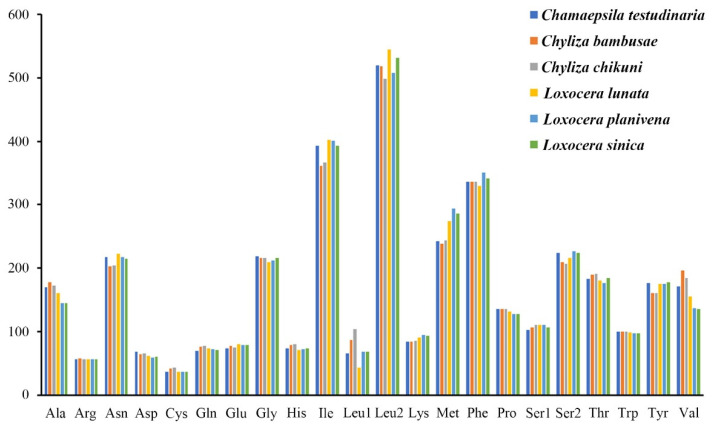
Patterns of codon usage of mitochondrial protein-coding genes of 6 Psilidae species. The *X*-axis shows the codon families, and the *Y*-axis shows the total codons.

**Figure 3:**
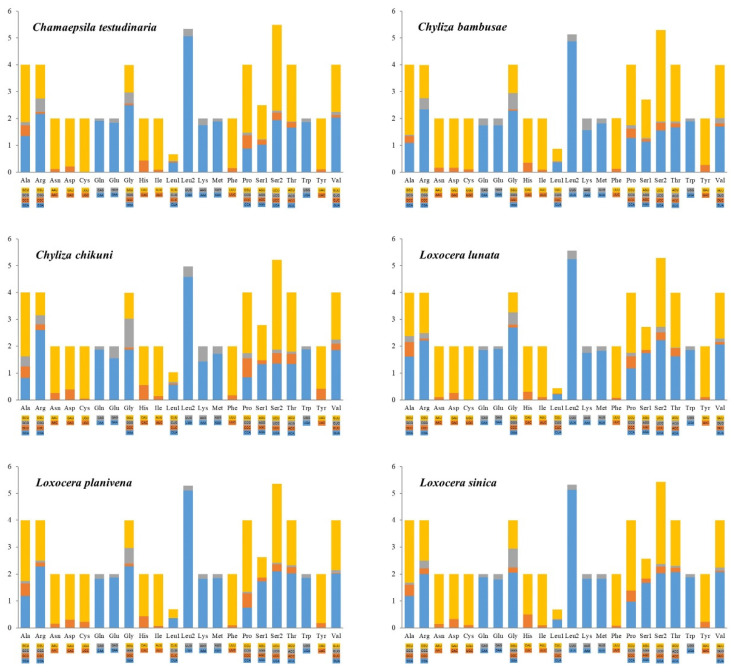
Relative synonymous codon usage (RSCU) of mitochondrial protein-coding genes of 6 Psilidae species. The *X*-axis shows different amino acids, and the *Y*-axis shows the RSCU value (the number of times a certain synonymous codon is used/the average number of times that all codons encoding the amino acid are used).

All 13 PCGs have the standard start codon ATN (ATT and ATG are the most frequently used), except that *COI* and *ND1* start with TCG and TTG in all the sampled rust flies, respectively. Start codons for *COI* are usually unregular in holometabolous insects [19], and TCG is one of the common start codons for dipteran *COI* [19,21,81]. The non-standard start codon TTG for *ND1* has also been found in several other mitogenomes of Diptera [82,83]. Each PCG is terminated with TAA or TAG as stop codons, or with a single T residue as an incomplete stop codon, which has been noticed in many other insect mitogenomes [19,21,72]. The incomplete stop codon is presumed to be filled by polyadenylation during the maturation of mRNA [84]. The most frequently used codon family is *trnL2* (>490), while the least is *trnC* (<50) in all the 6 mitogenomes (Figure 2). The relative synonymous codon usage (RSCU) patterns of the 6 mitogenomes are roughly the same, and the RSCU values are shown in Figure 3 with all possible synonymous codons of the 22 amino acids are presented. The most prevalently used codons are NNA and NNU for each amino acid (Figure 3).

**Figure 4:**
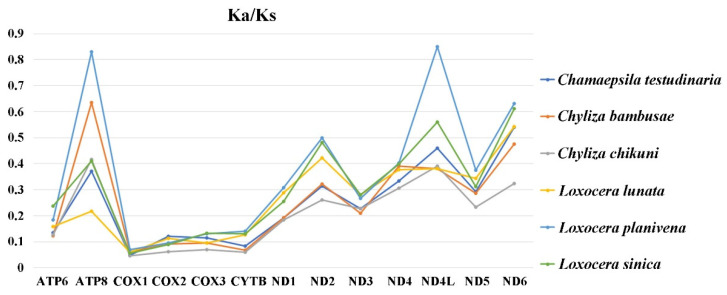
Evolutionary rates (ratios of Ka/Ks) of mitochondrial protein-coding genes of 6 Psilidae species. Abbreviations: ATP6 and ATP8 for adenosine triphosphate (ATP) synthase subunits 6 and 8; COX1–COX3 for cytochrome C oxidase subunits I–III; CYTB for cytochrome b; and ND1–ND6 and ND4L for nicotinamide adenine dinucleotide hydrogen (NADH) dehydrogenase subunits 1–6 and 4L.

The synonymous substitution rate (Ks) varies significantly among the 6 sampled species, while the non-synonymous substitution rates (Ka) is similar among them (Figure S1, Table S3). The ratio of Ka/Ks (ω) is a diagnostic statistic to detect molecular adaption [57,58] and is used to investigate the evolutionary rate of the PCGs. The ω values of the 13 PCGs of each species are shown in Figure 4. Species in Chylizinae exhibit a slower average evolutionary rate than species of Psilinae; the ω values of all 13 PCGs are lower than 1.0, indicating that they are under purifying selection [57,58]; *ATP8* (0.48), *ND4L* (0.504), and *ND6* (0.521) have very high evolutionary rates, while the ω value of *COI* (0.057) is the lowest (Figure 4, Table S3).

### 3.3. Transfer and Ribosomal RNA Genes

The typical set of 22 tRNAs were identified in all 6 psilid mitogenomes, ranging from 62 to 72 bp in length. The tRNAs exhibit high A + T content (77.3–80.1%), positive AT-skew and negative GC-skew (Table 2). All of tRNAs can be folded into the typical clover-leaf secondary structure except *trnS1*, which lacks the dihydrouridine (DHU) arm (Figure S2) as in many other insects [18,72,85]. Most arms of the tRNAs were formed by classical Watson-Crick base pairing, with 3 kinds of non-classical base pairing (G-T match, T-T match and A-A match) were found (Figure S2).

The *lrRNA* is located between *trnL1* and *trnV*, ranging from 1324 bp (*Chy. bambusae* and *Chy. chikuni*) to 1334 bp (*Cha. testudinaria* and *L. sinica*) in length. The *srRNA* is located between *trnV* and the control region, ranging from 790 bp (*Chy. chikuni* and *L. lunata*) to 797 bp (*Cha. testudinaria*). The rRNAs show high A + T bias with A + T content ranges from 81% to 83% (Table 2).

### 3.4. Control Region

**Figure 5:**
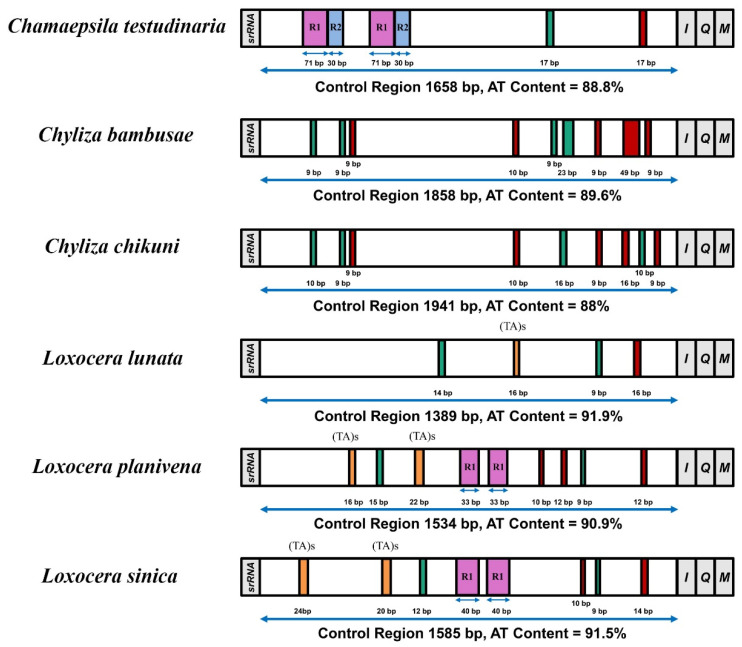
Control regions of mitochondrial genomes of 6 Psilidae species. Structure elements found in the control regions are labeled with different color blocks: repeat unit, pink and blue; poly-T, green; poly-A, red; (TA)s, orange; and control regions flanking genes *srRNA*, *trnI*, *trnQ,* and *trnM*, grey. R refers to repeat unit.

The control region (CR) is the longest non-coding region of the 6 Psilidae mitogenomes. The control regions are considerably variable in length, ranging from 1389 bp to 1941 bp, and appear much higher A + T content (88–91.9%) than the whole mitogenomes (Table 2). Several repeat sequences have been detected in 3 of the 6 Psilidae mitogenomes (Figure 5): 2 types of repeat units are found in the control region of *Cha. testudinaria*, whereas the control region of *L. planivena* and *L. sinica* each contains only a single type of repeat units. Besides, poly-A regions are found at the end of control region in all sampled species, and 4 poly-T and 5 poly-A regions are presented in the control region of the 2 species of Chylizinae (Figure 5). Furthermore, several microsatellite-like “(TA)_n_” units (16–24 bp) are found in the control region of the 3 *Loxocera* species (Figure 5). These simple sequence repeats (SSRs) have been considered as potential useful molecular markers in species identification, genetic diversity studies, and phylogenetic analyses [23,86]. The above results indicate that the length, the nucleotide compositions as well as the copy numbers of repeat units in the control regions are highly variable among the known Psilidae mitogenomes, and such structural differences may provide useful information for phylogenetic and evolutionary studies of rust flies. Besides, in all sampled species, an intergenic region over 25 bp in length is detected between *trnE* and *trnF*, with the longest one in *Cha. testudinaria* (103 bp).

### 3.5. Phylogenetic Analyses

**Figure 6:**
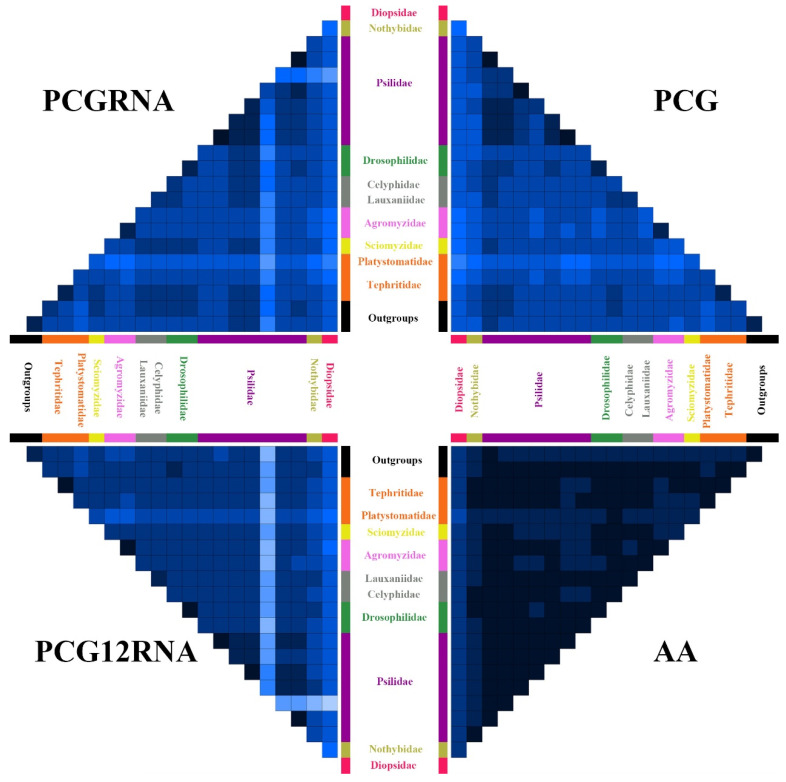
AliGROOVE analyses of AA, PCG, PCG12RNA, and PCGRNA datasets. The mean similarity score between sequences is represented by colored squares, based on AliGROOVE scores ranging from −1 [a great difference in rates from the remainder of the data set, or heterogeneity (red coloring)] to +1 [rates that matched all other comparisons (blue coloring)].

The sequence heterogeneity analyses show that the degrees of heterogeneity of the AA and PCG12RNA datasets are lower than those of the PCG and PCGRNA datasets (Figure 6). The lower heterogeneity of PCG12RNA dataset compared to the PCGRNA dataset indicate that third codon positions have higher heterogeneity than the first and second ones, as expected. The species of the family Diopsidae (*Teleopsis dalmanni* (Wiedemann)), Nothybidae (*Nothybus sumatranus* Enderlein) Nothybidae), and Platystomatidae (*Prosthiochaeta* sp.) exhibit a stronger heterogeneity in sequence divergence than to other acalyptrate species in all 4 datasets (Figure 6). The conspicuous high heterogeneity in sequence divergence of *Chamaepsila rosae* (Fabricius) (Psilidae) in the PCG12RNA and PCGRNA datasets (Figure 6) is attributed to a large amount of missing data in the rRNAs of the sequence. Highly heterogeneous sequences have been shown to reduce the nodal support confidence and topology accuracy [8,87,88], and the use of the heterogeneous model in phylogenetic analyses will largely improve the impact of heterogeneous sequences [9,89]. Therefore, the site-heterogeneous mixture CAT + GTR model was used in the phylogenetic analyses in this study.

**Figure 7:**
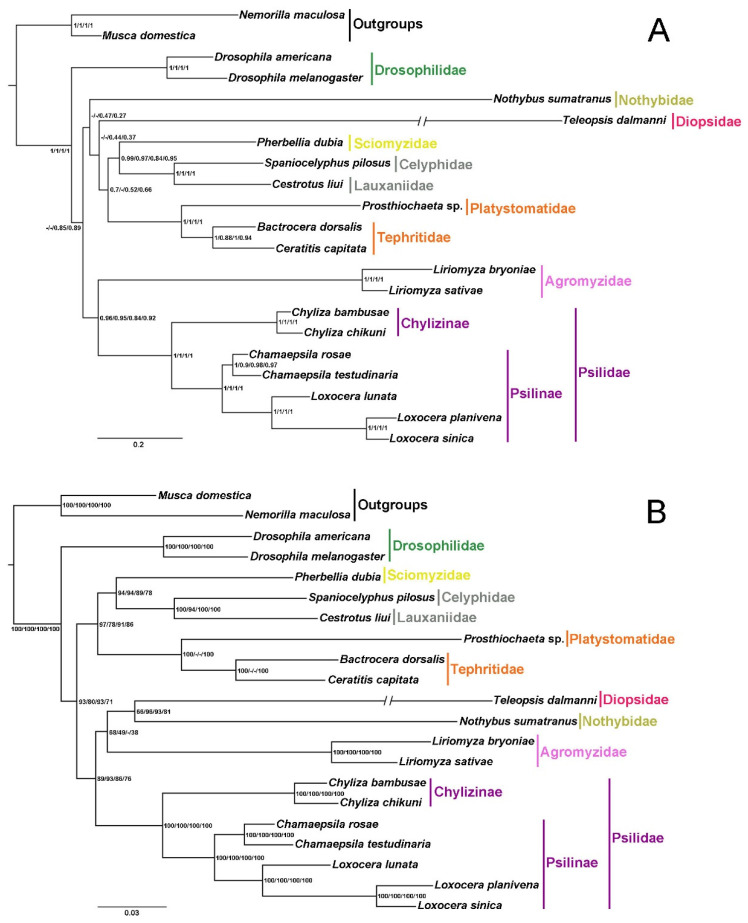
Phylogenetic trees inferred from Bayesian inference (**A**) and maximum likelihood (**B**) analyses of AA, PCG, PCG12RNA, and PCGRNA datasets. Supports at nodes (from left to right) are Bayesian posterior probabilities (BPP) or ML bootstrap values (BSV) for AA, PCG, PCG12RNA, and PCGRNA. “-” indicates node support values unavailable.

19 species from 10 acalyptrate families are included in the phylogenetic analyses, representing 6 of the 10 traditional acalyptrate superfamilies. Results reconstructed based on the 4 datasets present similar topologies regarding family-level relationships within Acalyptratae, but the positions of several branches appear to be ambiguous (Figure 7).

The sister relationship between Chylizinae and Psilinae is supported with high Bayesian posterior probabilities (BPP = 1) and ML bootstrap values (BSV = 100), forming the monophyletic Psilidae (Figure 7). Three subfamilies have been recognized within Psilidae to date, among them Chylizinae and Psilinae have long been considered to be putative sister groups [26,28]. The systematic position of the third subfamily Belobackenbardiinae, which contains 4 described species in a single genus *Belobackenbardia*, has remained controversial [26,41,90]. A recent morphology-based phylogenetic study of Diopsoidea recovered Belobackenbardiinae as the basal-most clade of the monophyletic Psilidae, sister to a clade formed by the extinct genus *Electrochyliza* and (Chylizinae + Psilinae) [28]. Psilinae is consistently divided into 2 major clades in the present study (BBP = 1, BSV = 100), one includes the species of *Chamaepsila* and the other the species of *Loxocera* (Figure 7). Species of Psilinae are mainly spilt into *Psila* s. lat. and *Loxocera* s. lat. [28,37,38,39], whereas some subgroups within the 2 genera are sometimes treated as separate genera [26,43]. Phylogenetic relationships of the subfamilies within Psilidae and the genus-level groups within Psilinae are in need of further study.

Most works have treated Psilidae as a member of the superfamily Diopsoidea following Hennig [91] and McAlpine [92]. This superfamily also includes Diopsidae, Nothybidae, and several other families, but its composition keeps changing [28]. Diopsoidea has been reviewed and redefined by Lonsdale [28] to include 7 families, and this point of view has been tested by a morphology-based phylogenetic analysis. However, the resulting topologies from all analyses in the present study indicate that Diopsoidea is not a monophyletic group, with Psilidae either forms sister groups with Agromyzidae or with ((Diopsidae + Nothybidae) + Agromyzidae), and the positions of Diopsidae and Nothybidae vary between different topologies (Figure 7). The non-monophyletic Diopsoidea has also been recovered in several phylogenetic studies based on morphology [93] and molecular data [25,94]. Nonetheless, the available molecular data for Diopsoidea are still very scarce. Considering that denser taxon sampling has been confirmed to greatly improve the accuracy of phylogenetic inferences [95], sequencing mitogenomes of more taxon of Diopsoidea could help investigating controversial taxonomic problems within the superfamily and resolving phylogeny within Acalyptratae.

## 4. Conclusions

The present study provides new data on the mitochondrial genomes of Psilidae, including the first 2 mitogenomes of the subfamily Chylizinae (*Chy. bambusae* and *Chy. chikuni*), 3 mitogenomes of the genus *Loxocera* (*L. lunata*, *L. planivena* and *L. sinica*), and 1 mitogenome of the genus *Chamaepsila* (*Cha.* *testudinaria*). Comparative analyses show that the psilid mitogenomes are conserved in structure and present putative ancestral gene arrangements; nucleotide composition of the 6 mitogenomes are distinctly Adenine plus Thymine biased; all 13 PCGs are initiated with ATN start codons, except for *COI* and *ND1* which started with TCG and TTG, respectively; TAA, TAG, or a single T residue are used as PCG stop codons; NNA and NNU are the most prevalently used codons for each amino acid; evolutionary rates vary among species, with species of Chylizinae exhibiting slower average rate than that of Psilinae; the length, the nucleotide composition, and the copy number of repeat units of the control region are highly variable among species, which may provide useful information for phylogenetic and evolutionary studies of Psilidae.

Bayesian and maximum likelihood analyses based on 4 datasets (AA, PCG, PCG12RNA, and PCGRNA) recover the monophyly of Psilidae, and the sister relationship between Chylizinae and Psilinae. Psilinae is divided into 2 major clades which represent *Loxocera* s. lat. and *Psila* s. lat., respectively. The monophyly of Diopsoidea is not supported in all the present analyses, with the position of Diopsidae and Nothybidae vary between different topologies, which may be due to the limited sampling of related taxa.

Our results show that the mitogenomic data are effective molecular markers to study the phylogeny and evolution of Psilidae, and sequencing mitogenomes of more taxa, especially the species of Belobackenbardiinae and Psilinae, could help to resolve the controversial taxonomic problems and higher-level phylogeny within the family.

## Figures and Tables

**Table 1:** Taxonomic information, GenBank accession numbers, and references of mitochondrial genomes used in the present study.

Superfamily	Family	Species	GenBank Number	Reference
Outgroup				
Muscoidea	Muscidae	*Musca domestica*	NC_024855	[59]
Oestroidea	Tachinidae	*Nemorilla maculosa*	MG786426	Direct submission
Ingroup				
Diopsoidea	Diopsidae	*Teleopsis dalmanni*	CM026973	Direct submission
	Nothybidae	*Nothybus sumatranus*	MW387954	[60]
	Psilidae	*Chamaepsila rosae*	MT941918	Direct submission
		*Chamaepsila testudinaria*	ON258616	Present study
		*Chyliza bambusae*	ON258617	Present study
		*Chyliza chikuni*	ON258618	Present study
		*Loxocera lunata*	ON258619	Present study
		*Loxocera planivena*	ON258620	Present study
		*Loxocera sinica*	ON258621	Present study
Ephydroidea	Drosophilidae	*Drosophila americana*	MK659804	Direct submission
		*Drosophila melanogaster*	NC_024511	Direct submission
Lauxanioidea	Celyphidae	*Spaniocelyphus pilosus*	KX372562	[61]
	Lauxaniidae	*Cestrotus liui*	KX372559	[61]
Opomyzoidea	Agromyzidae	*Liriomyza bryoniae*	JN570504	[62]
		*Liriomyza sativae*	JQ862475	[63]
Sciomyzoidea	Sciomyzidae	*Pherbellia dubia*	MT628567	Direct submission
Tephritoidea	Platystomatidae	*Prosthiochaeta* sp.	MT528242	[64]
	Tephritidae	*Bactrocera dorsalis*	KT343905	Direct submission
		*Ceratitis capitata*	NC_000857	[65]

**Table 2:** Nucleotide composition of mitochondrial genomes of the 6 Psilidae species.

Species	Regions	Length (bp)	T%	C%	A%	G%	A + T%	AT Skew	GC Skew
*Chamaepsila testudinaria*	Whole genome	16,609	37.3	12.2	41.5	9.1	78.8	0.053	−0.147
	PCGs	11,184	44.5	11.4	32.2	11.9	76.7	−0.16	0.023
	1st codon position	5537	37	12.6	41.6	8.8	78.6	0.058	−0.181
	2nd codon position	5536	39.9	10.5	42.2	7.4	82.1	0.028	−0.171
	3rd codon position	5536	35	13.4	40.6	11	75.6	0.075	−0.1
	tRNAs	1469	38.7	9.9	38.5	12.9	77.3	−0.003	0.132
	rRNAs	2131	43.7	6.3	38.2	11.7	81.9	−0.066	0.299
	Control region	1658	37.1	6.3	51.7	4.8	88.8	0.165	−0.135
*Chyliza bambusae*	Whole genome	16,664	36.7	13.1	41.6	8.6	78.3	0.062	−0.207
	PCGs	11,182	44.4	11.6	31.4	12.6	75.8	−0.172	0.041
	1st codon position	5555	38.5	12.5	42.4	6.5	81	0.048	−0.317
	2nd codon position	5555	35.2	13.1	41.5	10.2	76.7	0.081	−0.124
	3rd codon position	5554	36.3	13.7	40.8	9.1	77.2	0.058	−0.2
	tRNAs	1457	39	9.8	38.6	12.7	77.6	−0.005	0.132
	rRNAs	2116	43.1	6.1	38.4	12.5	81.5	−0.058	0.347
	Control region	1858	37.1	7.3	52.4	3.2	89.6	0.171	−0.392
*Chyliza chikuni*	Whole genome	16,759	36.5	14.1	40.5	9	77	0.052	−0.222
	PCGs	11,182	43.4	12.7	30.8	13.2	74.1	−0.17	0.019
	1st codon position	5587	36	13.7	40.8	9.5	76.9	0.062	−0.182
	2nd codon position	5586	36.8	14.4	39.5	9.3	76.3	0.035	−0.214
	3rd codon position	5586	36.6	14.2	41.2	8.1	77.8	0.059	−0.274
	tRNAs	1456	39	9.8	38.5	12.6	77.5	−0.006	0.125
	rRNAs	2114	42.8	6.4	38.3	12.6	81	−0.055	0.327
	Control region	1941	38.6	8.5	49.4	3.5	88	0.122	−0.416
*Loxocera lunata*	Whole genome	16,283	38.9	11.9	40.7	8.5	79.6	0.023	−0.165
	PCGs	11,196	43.8	10.9	33.6	11.7	77.4	−0.132	0.038
	1st codon position	5428	40.3	12.4	38.8	8.5	79.1	−0.02	−0.184
	2nd codon position	5428	39.2	11.3	41.2	8.3	80.4	0.025	−0.151
	3rd codon position	5427	37	12	42.2	8.8	79.2	0.065	−0.157
	tRNAs	1458	40.2	8.9	38.6	12.3	78.8	−0.02	0.165
	rRNAs	2118	43.5	5.9	39.3	11.3	82.8	−0.051	0.315
	Control region	1389	45.3	5.3	46.6	2.9	91.9	0.014	−0.292
*Loxocera planivena*	Whole genome	16,489	38.8	11.8	41.1	8.2	80	0.029	−0.177
	PCGs	11,183	44.3	11	33.5	11.2	77.7	−0.139	0.009
	1st codon position	5497	35	11.2	43.9	9.9	78.9	0.113	−0.059
	2nd codon position	5496	38.7	14.4	37.2	9.7	75.9	−0.02	−0.194
	3rd codon position	5496	42.8	9.8	42.3	5.1	85.1	−0.006	−0.318
	tRNAs	1458	39.7	8.6	40.2	11.5	79.9	0.006	0.147
	rRNAs	2123	42.7	5.9	40	11.4	82.7	−0.033	0.319
	Control region	1534	45.7	5.7	45.2	3.5	90.9	−0.006	−0.243
*Loxocera sinica*	Whole genome	16,527	38.8	11.8	41.2	8.3	80	0.031	−0.175
	PCGs	11,183	44.3	11.1	33.2	11.4	77.5	−0.143	0.013
	1st codon position	5509	38.4	10.4	42	9.1	80.5	0.045	−0.064
	2nd codon position	5509	37.2	14.4	38.7	9.8	75.9	0.02	−0.189
	3rd codon position	5509	40.7	10.5	43	5.8	83.6	0.027	−0.285
	tRNAs	1455	39.7	8.7	40.3	11.3	80.1	0.008	0.131
	rRNAs	2125	42.9	5.9	40.1	11.1	83	−0.034	0.308
	Control region	1585	44.5	5.2	46.9	3.3	91.5	0.026	−0.215

## Data Availability

All mitogenome sequences generated in this study were deposited in the GenBank under accession numbers ON258616 to ON258621.

## Supplementary Materials

The following are available online at https://www.mdpi.com/article/10.3390/insects13060518/s1, Figure S1: Synonymous (A) and non-synonymous (B) substitutional rates of mitochondrial protein-coding genes of 6 Psilidae species. Abbreviations: ATP6 and ATP8 for adenosine triphosphate (ATP) synthase subunits 6 and 8; COX1–COX3 for cytochrome C oxidase subunits I–III; CYTB for cytochrome b; ND1–ND6 and ND4L for nicotinamide adenine dinu-cleotide hydrogen (NADH) dehydrogenase subunits 1–6 and 4L., Figure S2: Inferred secondary structures of 22 tRNAs of 6 Psilidae species. The tRNAs are labeled with the abbreviations of their corresponding amino acids. Inferred Watson-Crick bonds are illustrated by lines and GU bonds are illustrated by dots. Other mismatches are indicated by blue arrows. Table S1: Information of the voucher specimens used for mitochondrial genomes sequencing in the present study, Table S2: Structure of *Chamaepsila testudinaria*, *Chyliza bambusae*, *Chyliza chikuni*, *Loxocera lunata*, *Loxocera planivena*, and *Loxocera sinica* mitochondrial genome, Table S3: Synonymous and non-synonymous substitutional analysis of gene ATP6, ATP8, COX1, COX2, COX3, CYTB, ND1, ND2, ND3, ND4, ND4L, ND5, ND6.
