# Supplementary material for: Mitochondrial Genomes Provide New Phylogenetic and Evolutionary Insights into Psilidae (Diptera: Brachycera)
Source: Insects. 2022 Jun 1;13(6):518. doi: 10.3390/insects13060518 (PMC9224655; doi:10.3390/insects13060518)
Supplement: Supplementary file 1 [file insects-13-00518-s001.zip › Table S1.pdf]

Table S1. Information of the voucher specimens used for mitochondrial genomes sequencing in the present study.

Subfamily	Species	Location	Collection Date	Voucher Number
Psilinae	*Chamaepsila testudinaria*	CHINA, Qinghai, Menyuan	2021.VII.2	ZJL-L07
Chylizinae	*Chyliza bambusae*	CHINA, Fujian, Wuyishan	2021.IV.16	ZJL-L11
Chylizinae	*Chyliza chikuni*	CHINA, Yunnan, Lvchun	2019.III.28	ZJL-L09
Psilinae	*Loxocera lunata*	CHINA, Yunnan, Lvchun	2019.III.26	ZJL-L04
Psilinae	*Loxocera planivena*	CHINA, Guangdong, Conghua	2020.X.18	ZJL-L05
Psilinae	*Loxocera sinica*	CHINA, Guangxi, Maoershan	2020.IX.3	ZJL-L02
