# Supplementary material for: Mitochondrial Genomes Provide New Phylogenetic and Evolutionary Insights into Psilidae (Diptera: Brachycera)
Source: Insects. 2022 Jun 1;13(6):518. doi: 10.3390/insects13060518 (PMC9224655; doi:10.3390/insects13060518)
Supplement: Supplementary file 1 [file insects-13-00518-s001.zip › Table S2.pdf]

Table S2-1. Structure of *Chamaepsila testudinaria* mitochondrial genome.

Gene	Direction	Location	Size	Anticodon	Codon		Intergenic nucleotides
					Start	Stop	
trnI	F	1–65	65	GAT			
trnQ	R	63–131	69	TTG			-3
trnM	F	139–207	69	CAT			7
ND2	F	208–1233	1026		ATT	TAA	0
trnW	F	1232–1299	68	TCA			-2
trnC	R	1292–1356	65	GCA			-8
trnY	R	1378–1443	66	GTA			21
COI	F	1442–2977	1536		TCG	TAA	-2
trnL2	F	2979–3043	65	TAA			1
COII	F	3045–3732	688		ATG	T-	1
trnK	F	3733–3803	71	CTT			0
trnD	F	3805–3871	67	GTC			1
ATP8	F	3872–4033	162		ATT	TAA	0
ATP6	F	4027–4704	678		ATG	TAA	-7
COIII	F	4704–5492	789		ATG	TAA	-1
trnG	F	5503–5569	67	TCC			10
ND3	F	5570–5923	354		ATT	TAA	0
trnA	F	5926–5990	65	TGC			2
trnR	F	5991–6053	63	TCG			0
trnN	F	6060–6124	65	GTT			6
trnS1	F	6125–6192	68	GCT			0
trnE	F	6195–6261	67	TTC			2
trnF	R	6365–6431	67	GAA			103
ND5	R	6431–8140	1710		ATT	TAA	-1
trnH	R	8165–8229	65	GTG			24
ND4	R	8230–9568	1339		ATG	T-	0
ND4L	R	9562–9858	297		ATG	TAA	-7
trnT	F	9861–9924	64	TGT			2
trnP	R	9925–9991	67	TGG			0
ND6	F	9994–10515	522		ATT	TAA	2
CytB	F	10515–11651	1137		ATG	TAG	-1
trnS2	F	11650–11716	67	TGA			-2
ND1	R	11735–12680	946		TTG	T-	18
trnL1	R	12682–12748	67	TAG			1
lrRNA	R	12749–14082	1334				0
trnV	R	14083–14154	72	TAC			0
srRNA	R	14155–14951	797				0
control region	F	14952–16609	1658				0

Table S2-2. Structure of *Chyliza bambusae* mitochondrial genome.

Gene	Direction	Location	Size	Anticodon	Codon		Intergenic nucleotides
					Start	Stop	
trnI	F	1–65	65	GAT			
trnQ	R	63–131	69	TTG			-3
trnM	F	131–199	69	CAT			-1
ND2	F	200–1225	1026		ATT	TAA	0
trnW	F	1224–1291	68	TCA			-2
trnC	R	1284–1346	63	GCA			-8
trnY	R	1347–1412	66	GTA			0
COI	F	1411–2946	1536		TCG	TAA	-2
trnL2	F	2947–3011	65	TAA			0
COII	F	3014–3701	688		ATG	T-	2
trnK	F	3702–3771	70	CTT			0
trnD	F	3772–3837	66	GTC			0
ATP8	F	3838–3996	159		ATC	TAA	0
ATP6	F	3990–4667	678		ATG	TAA	-7
COIII	F	4667–5455	789		ATG	TAA	-1
trnG	F	5464–5528	65	TCC			8
ND3	F	5529–5882	354		ATA	TAA	0
trnA	F	5883–5945	63	TGC			0
trnR	F	5946–6008	63	TCG			0
trnN	F	6010–6075	66	GTT			1
trnS1	F	6076–6143	68	GCT			0
trnE	F	6145–6210	66	TTC			1
trnF	R	6244–6309	66	GAA			33
ND5	R	6310–8020	1711		ATT	T-	0
trnH	R	8036–8099	64	GTG			15
ND4	R	8100–9438	1339		GTG	T-	0
ND4L	R	9432–9728	297		ATG	TAA	-7
trnT	F	9731–9795	65	TGT			2
trnP	R	9796–9861	66	TGG			0
ND6	F	9864–10385	522		ATT	TAA	2
CytB	F	10385–11521	1137		ATG	TAG	-1
trnS2	F	11520–11586	67	TGA			-2
ND1	R	11607–12552	946		TTG	T-	20
trnL1	R	12554–12618	65	TAG			1
lrRNA	R	12619–13942	1324				0
trnV	R	13943–14014	72	TAC			0
srRNA	R	14015–14806	792				0
control region	F	14807–16664	1858				0

Table S2-3. Structure of *Chyliza chikuni* mitochondrial genome.

Gene	Direction	Location	Size	Anticodon	Codon		Intergenic nucleotides
					Start	Stop	
trnI	F	1–65	65	GAT			
trnQ	R	63–131	69	TTG			-3
trnM	F	131–199	69	CAT			-1
ND2	F	200–1225	1026		ATT	TAA	0
trnW	F	1226–1293	68	TCA			0
trnC	R	1286–1348	63	GCA			-8
trnY	R	1362–1427	66	GTA			13
COI	F	1426–2961	1536		TCG	TAA	-2
trnL2	F	2962–3026	65	TAA			0
COII	F	3029–3716	688		ATG	T-	2
trnK	F	3717–3786	70	CTT			0
trnD	F	3787–3852	66	GTC			0
ATP8	F	3853–4011	159		ATT	TAA	0
ATP6	F	4005–4682	678		ATG	TAA	-7
COIII	F	4682–5470	789		ATG	TAA	-1
trnG	F	5479–5543	65	TCC			8
ND3	F	5544–5897	354		ATT	TAA	0
trnA	F	5899–5961	63	TGC			1
trnR	F	5962–6024	63	TCG			0
trnN	F	6025–6089	65	GTT			0
trnS1	F	6090–6157	68	GCT			0
trnE	F	6159–6225	67	TTC			1
trnF	R	6260–6325	66	GAA			34
ND5	R	6326–8036	1711		ATT	T-	0
trnH	R	8052–8115	64	GTG			15
ND4	R	8116–9454	1339		GTG	T-	0
ND4L	R	9448–9744	297		ATG	TAA	-7
trnT	F	9747–9811	65	TGT			2
trnP	R	9812–9876	65	TGG			0
ND6	F	9879–10400	522		ATT	TAA	2
CytB	F	10400–11536	1137		ATG	TAG	-1
trnS2	F	11535–11601	67	TGA			-2
ND1	R	11621–12566	946		TTG	T-	19
trnL1	R	12568–12632	65	TAG			1
lrRNA	R	12633–13956	1324				0
trnV	R	13957–14028	72	TAC			0
srRNA	R	14029–14818	790				0
control region	F	14819–16759	1941				0

Table S2-4. Structure of *Loxocera lunata* mitochondrial genome.

Gene	Direction	Location	Size	Anticodon	Codon		Intergenic nucleotides
					Start	Stop	
trnI	F	1–65	65	GAT			
trnQ	R	89–157	69	TTG			23
trnM	F	165–233	69	CAT			7
ND2	F	234–1259	1026		ATT	TAA	0
trnW	F	1258–1324	67	TCA			-2
trnC	R	1317–1379	63	GCA			-8
trnY	R	1387–1451	65	GTA			7
COI	F	1450–2985	1536		TCG	TAA	-2
trnL2	F	3024–3088	65	TAA			38
COII	F	3089–3773	685		ATG	T-	0
trnK	F	3774–3843	70	CTT			0
trnD	F	3844–3909	66	GTC			0
ATP8	F	3910–4074	165		ATT	TAA	0
ATP6	F	4068–4745	678		ATG	TAA	-7
COIII	F	4745–5533	789		ATG	TAA	-1
trnG	F	5540–5604	65	TCC			6
ND3	F	5605–5958	354		ATT	TAA	0
trnA	F	5961–6024	64	TGC			2
trnR	F	6025–6088	64	TCG			0
trnN	F	6092–6156	65	GTT			3
trnS1	F	6157–6224	68	GCT			0
trnE	F	6227–6292	66	TTC			2
trnF	R	6320–6385	66	GAA			27
ND5	R	6385–8106	1722		ATT	TAA	-1
trnH	R	8122–8187	66	GTG			15
ND4	R	8188–9526	1339		ATG	T-	0
ND4L	R	9520–9816	297		ATG	TAA	-7
trnT	F	9819–9883	65	TGT			2
trnP	R	9884–9949	66	TGG			0
ND6	F	9952–10473	522		ATT	TAA	2
CytB	F	10473–11609	1137		ATG	TAG	-1
trnS2	F	11608–11673	66	TGA			-2
ND1	R	11692–12637	946		TTG	T-	18
trnL1	R	12639–12704	66	TAG			1
lrRNA	R	12705–14032	1328				0
trnV	R	14033–14104	72	TAC			0
srRNA	R	14105–14894	790				0
control region	F	14895–16283	1389				0

Table S2-5. Structure of *Loxocera planivena* mitochondrial genome.

Gene	Direction	Location	Size	Anticodon	Codon		Intergenic nucleotides
					Start	Stop	
trnI	F	1–65	65	GAT			
trnQ	R	66–134	69	TTG			0
trnM	F	134–201	68	CAT			-1
ND2	F	202–1227	1026		ATT	TAA	0
trnW	F	1226–1293	68	TCA			-2
trnC	R	1286–1349	64	GCA			-8
trnY	R	1421–1485	65	GTA			71
COI	F	1484–3019	1536		TCG	TAA	-2
trnL2	F	3081–3144	64	TAA			61
COII	F	3145–3826	682		ATG	T-	0
trnK	F	3827–3896	70	CTT			0
trnD	F	3897–3962	66	GTC			0
ATP8	F	3963–4124	162		ATA	TAA	0
ATP6	F	4118–4795	678		ATG	TAA	-7
COIII	F	4795–5583	789		ATG	TAA	-1
trnG	F	5590–5654	65	TCC			6
ND3	F	5655–6008	354		ATT	TAA	0
trnA	F	6011–6073	63	TGC			2
trnR	F	6073–6135	63	TCG			-1
trnN	F	6135–6201	67	GTT			-1
trnS1	F	6202–6268	67	GCT			0
trnE	F	6271–6335	65	TTC			2
trnF	R	6376–6444	69	GAA			40
ND5	R	6444–8162	1719		ATT	TAA	-1
trnH	R	8178–8242	65	GTG			15
ND4	R	8243–9581	1339		ATG	T-	0
ND4L	R	9582–9872	291		ATG	TAA	0
trnT	F	9875–9940	66	TGT			2
trnP	R	9941–10004	64	TGG			0
ND6	F	10007–10528	522		ATT	TAA	2
CytB	F	10528–11664	1137		ATG	TAG	-1
trnS2	F	11663–11728	66	TGA			-2
ND1	R	11745–12692	948		TTG	TAA	16
trnL1	R	12694–12760	67	TAG			1
lrRNA	R	12761–14092	1332				0
trnV	R	14093–14164	72	TAC			0
srRNA	R	14165–14955	791				0
control region	F	14956–16489	1534				0

Table S2-6. Structure of *Loxocera sinica* mitochondrial genome.

Gene	Direction	Location	Size	Anticodon	Codon		Intergenic nucleotides
					Start	Stop	
trnI	F	1–65	65	GAT			
trnQ	R	66–134	69	TTG			0
trnM	F	134–201	68	CAT			-1
ND2	F	202–1227	1026		ATT	TAA	0
trnW	F	1226–1293	68	TCA			-2
trnC	R	1286–1349	64	GCA			-8
trnY	R	1423–1487	65	GTA			73
COI	F	1486–3021	1536		TCG	TAA	-2
trnL2	F	3064–3128	65	TAA			42
COII	F	3129–3810	682		ATG	T-	0
trnK	F	3811–3881	71	CTT			0
trnD	F	3885–3950	66	GTC			3
ATP8	F	3951–4112	162		ATA	TAA	0
ATP6	F	4106–4783	678		ATG	TAA	-7
COIII	F	4783–5571	789		ATG	TAA	-1
trnG	F	5578–5642	65	TCC			6
ND3	F	5643–5996	354		ATA	TAA	0
trnA	F	5999–6060	62	TGC			2
trnR	F	6061–6123	63	TCG			0
trnN	F	6124–6189	66	GTT			0
trnS1	F	6190–6256	67	GCT			0
trnE	F	6259–6323	65	TTC			2
trnF	R	6364–6430	67	GAA			40
ND5	R	6430–8148	1719		ATT	TAA	-1
trnH	R	8164–8228	65	GTG			15
ND4	R	8229–9567	1339		ATG	T-	0
ND4L	R	9568–9858	291		ATG	TAA	0
trnT	F	9861–9924	64	TGT			2
trnP	R	9925–9989	65	TGG			0
ND6	F	9992–10513	522		ATT	TAA	2
CytB	F	10513–11649	1137		ATG	TAG	-1
trnS2	F	11648–11713	66	TGA			-2
ND1	R	11730–12677	948		TTG	TAA	16
trnL1	R	12679–12745	67	TAG			1
lrRNA	R	12746–14079	1334				0
trnV	R	14080–14151	72	TAC			0
srRNA	R	14152–14942	791				0
control region	F	14943–16527	1585				0
