# Supplementary material for: Mitochondrial Genomes Provide New Phylogenetic and Evolutionary Insights into Psilidae (Diptera: Brachycera)
Source: Insects. 2022 Jun 1;13(6):518. doi: 10.3390/insects13060518 (PMC9224655; doi:10.3390/insects13060518)
Supplement: Supplementary file 1 [file insects-13-00518-s001.zip › Table S3.pdf]

Table S3-1. Synonymous and non-synonymous substitutional analysis of gene ATP6.

Species	SynDif	SynPos	Ks	NSynDif	NSynPos	Ka	Ka/Ks
Chamaepsila testudinaria	72.67	153	0.7523	49.33	522	0.101	0.134254951
Chyliza bambusae	72.33	155.67	0.7248	43.67	519.33	0.0892	0.123068433
Chyliza chikuni	75.33	156.67	0.7686	47.67	518.33	0.0981	0.12763466
Loxocera lunata	71.17	153.17	0.7247	55.83	521.83	0.1154	0.159238306
Loxocera planivena	68.17	152.83	0.6773	59.83	522.17	0.1243	0.183522811
Loxocera sinica	56.83	151.83	0.5185	59.17	523.17	0.1226	0.236451302

Abbreviations: SynDif, the total number of synonymous differences; SynPos, the total number of synonymous sites; Ks, the number of synonymous (or silent) substitutions per synonymous (or silent) site; NSynDif, the total number of non-synonymous differences; NSynPos, the total number of non-synonymous sites; Ka, the number of non-synonymous substitutions per non-synonymous site.

Table S3-2. Synonymous and non-synonymous substitutional analysis of gene ATP8.

Species	SynDif	SynPos	Ks	NSynDif	NSynPos	Ka	Ka/Ks
Chamaepsila testudinaria	6.75	24.50	0.3434	12.25	104.5	0.1275	0.371287129
Chyliza bambusae	8.33	25.50	0.4292	23.67	103.5	0.2728	0.635601118
Chyliza chikuni	10.00	25.17	0.5659	21.00	103.83	0.2357	0.416504683
Loxocera lunata	11.75	24.00	0.7933	16.25	105	0.1733	0.218454557
Loxocera planivena	5.92	25.00	0.2844	21.08	104	0.2363	0.830872011
Loxocera sinica	8.75	24.50	0.485	18.25	104.5	0.1988	0.409896907

Abbreviations: SynDif, the total number of synonymous differences; SynPos, the total number of synonymous sites; Ks, the number of synonymous (or silent) substitutions per synonymous (or silent) site; NSynDif, the total number of non-synonymous differences; NSynPos, the total number of non-synonymous sites; Ka, the number of non-synonymous substitutions per non-synonymous site.

Table S3-3. Synonymous and non-synonymous substitutional analysis of gene COX1.

Species	SynDif	SynPos	Ks	NSynDif	NSynPos	Ka	Ka/Ks
Chamaepsila testudinaria	166.42	352.83	0.7434	40.58	1141.17	0.0364	0.048964218
Chyliza bambusae	162.67	352.83	0.7153	49.33	1141.17	0.0445	0.062211659
Chyliza chikuni	191.92	353.5	0.9652	50.08	1140.5	0.0453	0.046933278
Loxocera lunata	172.42	350.33	0.8008	52.58	1143.67	0.0474	0.059190809
Loxocera planivena	178.08	349.5	0.8531	65.92	1144.5	0.0599	0.070214512
Loxocera sinica	193.42	350.83	0.9962	60.58	1143.17	0.055	0.055209797

Abbreviations: SynDif, the total number of synonymous differences; SynPos, the total number of synonymous sites; Ks, the number of synonymous (or silent) substitutions per synonymous (or silent) site; NSynDif, the total number of non-synonymous differences; NSynPos, the total number of non-synonymous sites; Ka, the number of non-synonymous substitutions per non-synonymous site.

Table S3-4. Synonymous and non-synonymous substitutional analysis of gene COX2.

Species	SynDif	SynPos	Ks	NSynDif	NSynPos	Ka	Ka/Ks
Chamaepsila testudinaria	58.67	144	0.5876	36.33	537	0.0709	0.120660313
Chyliza bambusae	73.00	147.33	0.8105	38.00	533.67	0.0748	0.092288711
Chyliza chikuni	87.83	147	1.1947	37.17	534	0.073	0.061103206
Loxocera lunata	60.83	143.67	0.6236	36.17	537.33	0.0705	0.113053239
Loxocera planivena	76.83	144.17	0.93	45.17	536.83	0.0892	0.095913978
Loxocera sinica	76.50	143.33	0.9326	42.50	537.67	0.0835	0.089534634

Abbreviations: SynDif, the total number of synonymous differences; SynPos, the total number of synonymous sites; Ks, the number of synonymous (or silent) substitutions per synonymous (or silent) site; NSynDif, the total number of non-synonymous differences; NSynPos, the total number of non-synonymous sites; Ka, the number of non-synonymous substitutions per non-synonymous site.

Table S3-5. Synonymous and non-synonymous substitutional analysis of gene COX3.

Species	SynDif	SynPos	Ks	NSynDif	NSynPos	Ka	Ka/Ks
Chamaepsila testudinaria	85.50	179.83	0.7537	49.50	606.17	0.0865	0.114767149
Chyliza bambusae	79.17	179.33	0.6661	36.83	606.67	0.0633	0.095030776
Chyliza chikuni	93.67	180.5	0.883	36.33	605.5	0.0625	0.070781427
Loxocera lunata	89.00	176.83	0.8339	46.00	609.17	0.0796	0.095455091
Loxocera planivena	89.83	176.17	0.8544	63.17	609.83	0.1115	0.130500936
Loxocera sinica	93.33	177.17	0.909	67.67	608.83	0.1203	0.132343234

Abbreviations: SynDif, the total number of synonymous differences; SynPos, the total number of synonymous sites; Ks, the number of synonymous (or silent) substitutions per synonymous (or silent) site; NSynDif, the total number of non-synonymous differences; NSynPos, the total number of non-synonymous sites; Ka, the number of non-synonymous substitutions per non-synonymous site.

Table S3-6. Synonymous and non-synonymous substitutional analysis of gene CYTB.

Species	SynDif	SynPos	Ks	NSynDif	NSynPos	Ka	Ka/Ks
Chamaepsila testudinaria	124.33	253.17	0.7978	55.67	880.83	0.066	0.082727501
Chyliza bambusae	141	256.17	0.9929	57.00	877.83	0.0679	0.068385537
Chyliza chikuni	136.33	253	0.9507	47.67	881	0.0562	0.059114337
Loxocera lunata	113.5	253.33	0.6823	72.50	880.67	0.0872	0.127803019
Loxocera planivena	120.5	250.67	0.7682	88.50	883.33	0.1075	0.139937516
Loxocera sinica	119.17	249.83	0.7579	81.83	884.17	0.0988	0.130360206

Abbreviations: SynDif, the total number of synonymous differences; SynPos, the total number of synonymous sites; Ks, the number of synonymous (or silent) substitutions per synonymous (or silent) site; NSynDif, the total number of non-synonymous differences; NSynPos, the total number of non-synonymous sites; Ka, the number of non-synonymous substitutions per non-synonymous site.

Table S3-7. Synonymous and non-synonymous substitutional analysis of gene ND1.

Species	SynDif	SynPos	Ks	NSynDif	NSynPos	Ka	Ka/Ks
Chamaepsila testudinaria	81.83	207.33	0.5603	74.17	737.67	0.108	0.192753882
Chyliza bambusae	74.83	206.67	0.4945	66.17	738.33	0.0954	0.192922144
Chyliza chikuni	82.00	207.5	0.5613	71.00	737.5	0.103	0.183502583
Loxocera lunata	68.50	206.83	0.437	85.50	738.17	0.1258	0.287871854
Loxocera planivena	68.33	204.33	0.4428	92.67	740.67	0.1369	0.309168925
Loxocera sinica	76.67	205.5	0.516	89.33	739.5	0.1317	0.255232558

Abbreviations: SynDif, the total number of synonymous differences; SynPos, the total number of synonymous sites; Ks, the number of synonymous (or silent) substitutions per synonymous (or silent) site; NSynDif, the total number of non-synonymous differences; NSynPos, the total number of non-synonymous sites; Ka, the number of non-synonymous substitutions per non-synonymous site.

Table S3-8. Synonymous and non-synonymous substitutional analysis of gene ND2.

Species	SynDif	SynPos	Ks	NSynDif	NSynPos	Ka	Ka/Ks
Chamaepsila testudinaria	91.58	201.5	0.6986	143.42	752.5	0.2199	0.314772402
Chyliza bambusae	93.50	199.33	0.7365	153.5	754.67	0.2373	0.322199593
Chyliza chikuni	103.33	200.5	0.8716	147.67	753.5	0.2271	0.260555301
Loxocera lunata	75.25	199.67	0.5236	144.75	754.33	0.2216	0.423223835
Loxocera planivena	72.17	197.67	0.5003	160.83	756.33	0.2501	0.49990006
Loxocera sinica	74.83	199.17	0.5213	161.17	754.83	0.2513	0.482064071

Abbreviations: SynDif, the total number of synonymous differences; SynPos, the total number of synonymous sites; Ks, the number

of synonymous (or silent) substitutions per synonymous (or silent) site; NSynDif, the total number of non-synonymous differences; NSynPos, the total number of non-synonymous sites; Ka, the number of non-synonymous substitutions per non-synonymous site.

Table S3-9. Synonymous and non-synonymous substitutional analysis of gene ND3.

Species	SynDif	SynPos	Ks	NSynDif	NSynPos	Ka	Ka/Ks
Chamaepsila testudinaria	29.33	71.50	0.5939	34.67	279.5	0.1356	0.228321266
Chyliza bambusae	35.83	72.67	0.8036	42.17	278.33	0.1692	0.210552514
Chyliza chikuni	35.33	72.33	0.7902	44.67	278.67	0.1803	0.228170084
Loxocera lunata	29.33	72.83	0.5775	40.67	278.17	0.1626	0.281558442
Loxocera planivena	30.33	71.67	0.6232	41.67	279.33	0.1663	0.266848524
Loxocera sinica	29.83	72.00	0.603	42.17	279	0.1688	0.279933665

Abbreviations: SynDif, the total number of synonymous differences; SynPos, the total number of synonymous sites; Ks, the number of synonymous (or silent) substitutions per synonymous (or silent) site; NSynDif, the total number of non-synonymous differences; NSynPos, the total number of non-synonymous sites; Ka, the number of non-synonymous substitutions per non-synonymous site.

Table S3-10. Synonymous and non-synonymous substitutional analysis of gene ND4.

Species	SynDif	SynPos	Ks	NSynDif	NSynPos	Ka	Ka/Ks
Chamaepsila testudinaria	101.58	293.5	0.4642	146.42	1044.5	0.1552	0.334338647
Chyliza bambusae	94.25	295.67	0.4151	151.75	1042.33	0.1619	0.3900265
Chyliza chikuni	111.92	296.67	0.5244	150.08	1041.33	0.1601	0.305301297
Loxocera lunata	94.17	291.67	0.4222	149.83	1046.33	0.1589	0.376361914
Loxocera planivena	98.08	292.17	0.4451	166.92	1045.83	0.1795	0.403280162
Loxocera sinica	96.33	292.17	0.4344	161.67	1045.83	0.1731	0.398480663

Abbreviations: SynDif, the total number of synonymous differences; SynPos, the total number of synonymous sites; Ks, the number of synonymous (or silent) substitutions per synonymous (or silent) site; NSynDif, the total number of non-synonymous differences; NSynPos, the total number of non-synonymous sites; Ka, the number of non-synonymous substitutions per non-synonymous site.

Table S3-11. Synonymous and non-synonymous substitutional analysis of gene ND4L.

Species	SynDif	SynPos	Ks	NSynDif	NSynPos	Ka	Ka/Ks
Chamaepsila testudinaria	19.50	62.00	0.4077	37.50	226	0.1875	0.459896983
Chyliza bambusae	21.83	61.17	0.4846	37.17	226.83	0.1849	0.381551795
Chyliza chikuni	21.67	61.50	0.4758	37.33	226.5	0.1861	0.391130727
Loxocera lunata	19.83	59.83	0.4375	34.17	228.17	0.167	0.381714286
Loxocera planivena	11.33	59.67	0.219	37.67	228.33	0.1863	0.850684932
Loxocera sinica	15.33	60.00	0.3125	35.67	228	0.1754	0.56128

Abbreviations: SynDif, the total number of synonymous differences; SynPos, the total number of synonymous sites; Ks, the number of synonymous (or silent) substitutions per synonymous (or silent) site; NSynDif, the total number of non-synonymous differences; NSynPos, the total number of non-synonymous sites; Ka, the number of non-synonymous substitutions per non-synonymous site.

Table S3-12. Synonymous and non-synonymous substitutional analysis of gene ND5.

Species	SynDif	SynPos	Ks	NSynDif	NSynPos	Ka	Ka/Ks
Chamaepsila testudinaria	126.33	355.67	0.4813	162.67	1267.33	0.1408	0.292541035
Chyliza bambusae	129.67	358	0.4947	163.33	1265	0.1417	0.286436224
Chyliza chikuni	152.5	356.33	0.6341	169.5	1266.67	0.1474	0.232455449
Loxocera lunata	124.33	350.5	0.4804	188.67	1272.5	0.1652	0.3438801
Loxocera planivena	114.83	353.33	0.426	183.17	1269.67	0.1602	0.376056338

Loxocera sinica	132.08	354.5	0.5151	183.92	1268.5	0.1611	0.312754805

Abbreviations: SynDif, the total number of synonymous differences; SynPos, the total number of synonymous sites; Ks, the number of synonymous (or silent) substitutions per synonymous (or silent) site; NSynDif, the total number of non-synonymous differences; NSynPos, the total number of non-synonymous sites; Ka, the number of non-synonymous substitutions per non-synonymous site.

Table S3-13. Synonymous and non-synonymous substitutional analysis of gene ND6.

Species	SynDif	SynPos	Ks	NSynDif	NSynPos	Ka	Ka/Ks
Chamaepsila testudinaria	35.67	101.67	0.473	81.33	375.33	0.2557	0.540591966
Chyliza bambusae	39.25	100.17	0.5543	83.75	376.83	0.2636	0.475554754
Chyliza chikuni	47.83	100.83	0.7508	78.17	376.17	0.2433	0.324054342
Loxocera lunata	34.50	99.00	0.4686	81.50	378	0.2542	0.542466923
Loxocera planivena	37.00	99.83	0.5111	99.00	377.17	0.3231	0.632165917
Loxocera sinica	39.00	101.5	0.5386	100	375.5	0.329	0.610842926

Abbreviations: SynDif, the total number of synonymous differences; SynPos, the total number of synonymous sites; Ks, the number of synonymous (or silent) substitutions per synonymous (or silent) site; NSynDif, the total number of non-synonymous differences; NSynPos, the total number of non-synonymous sites; Ka, the number of non-synonymous substitutions per non-synonymous site.
