# Supplementary figures and images for: Mitochondrial Genomes Provide New Phylogenetic and Evolutionary Insights into Psilidae (Diptera: Brachycera)
Source: Insects. 2022 Jun 1;13(6):518. doi: 10.3390/insects13060518 (PMC9224655; doi:10.3390/insects13060518)

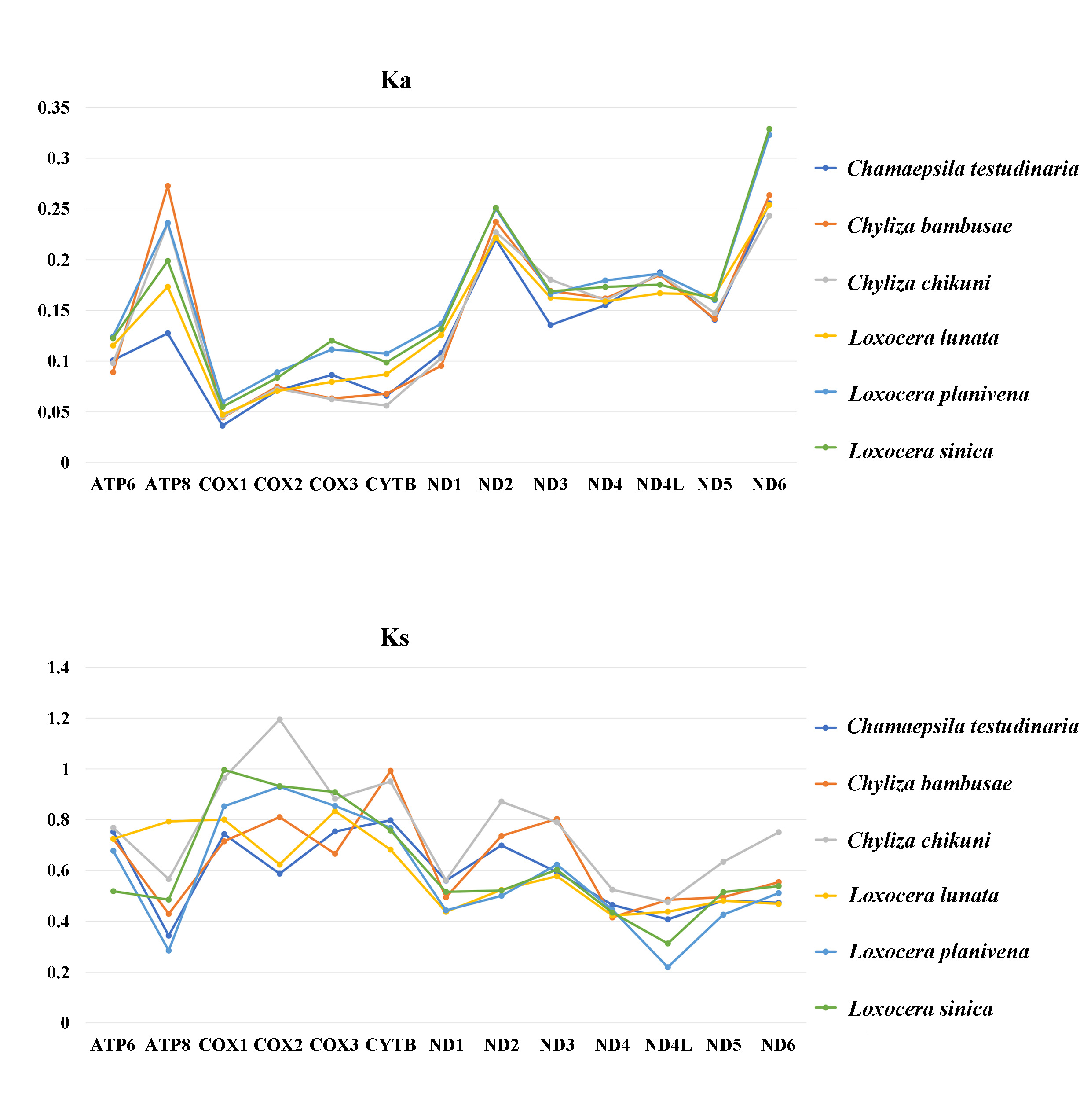

Supplement: Supplementary file 1 [file insects-13-00518-s001.zip › Figure S1.jpg]

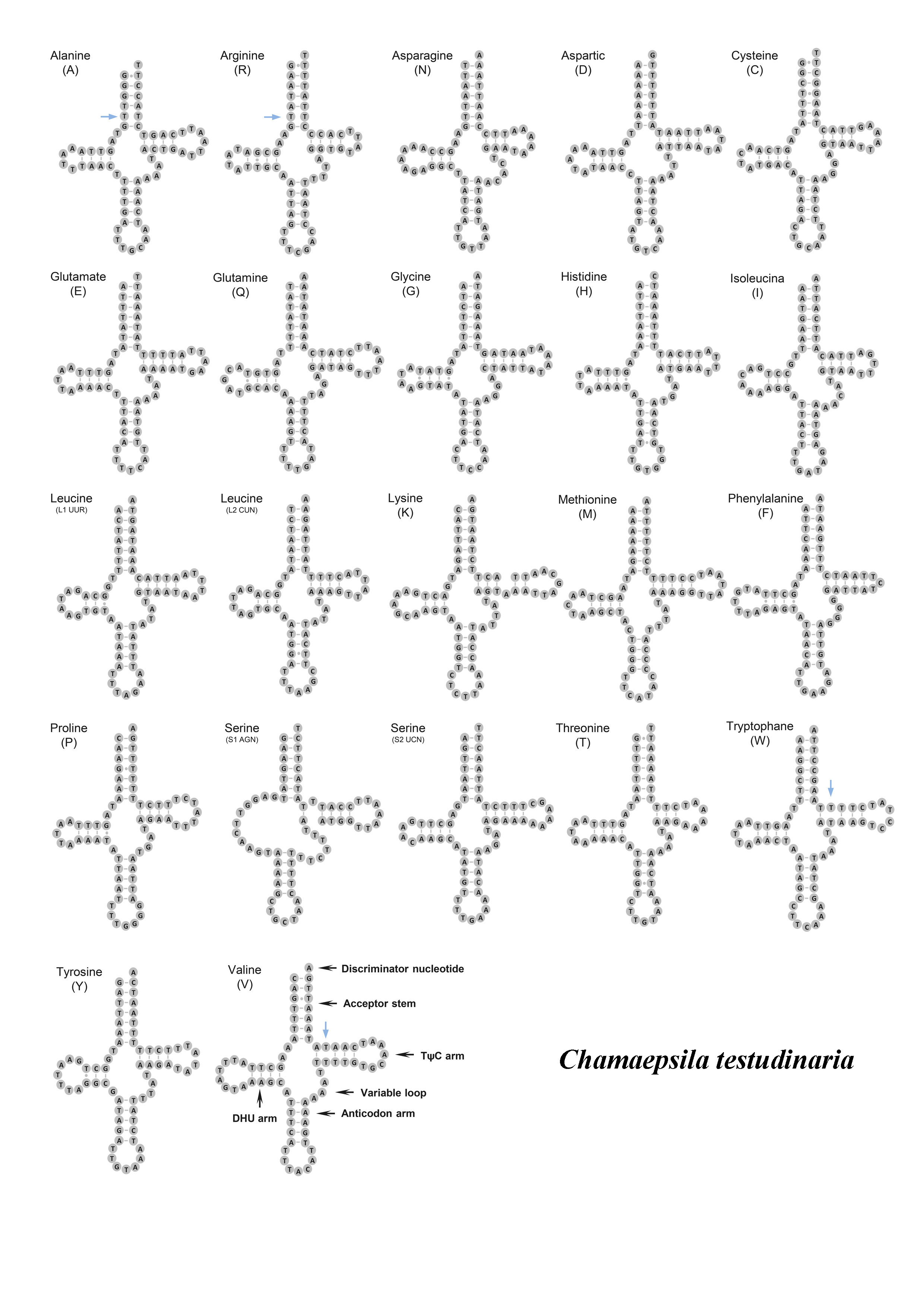

Supplement: Supplementary file 1 [file insects-13-00518-s001.zip › Figure S2-1.jpg]

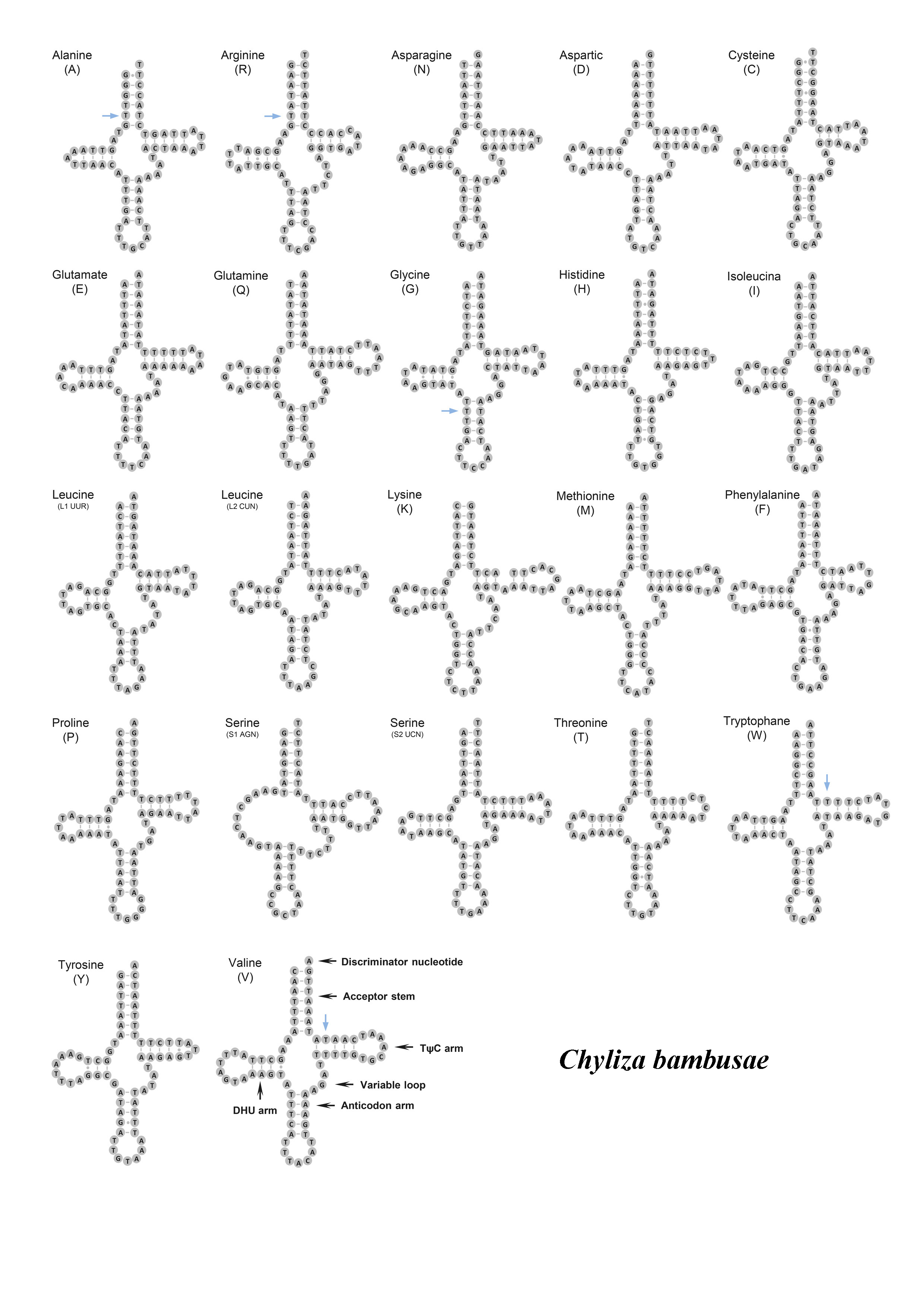

Supplement: Supplementary file 1 [file insects-13-00518-s001.zip › Figure S2-2.jpg]

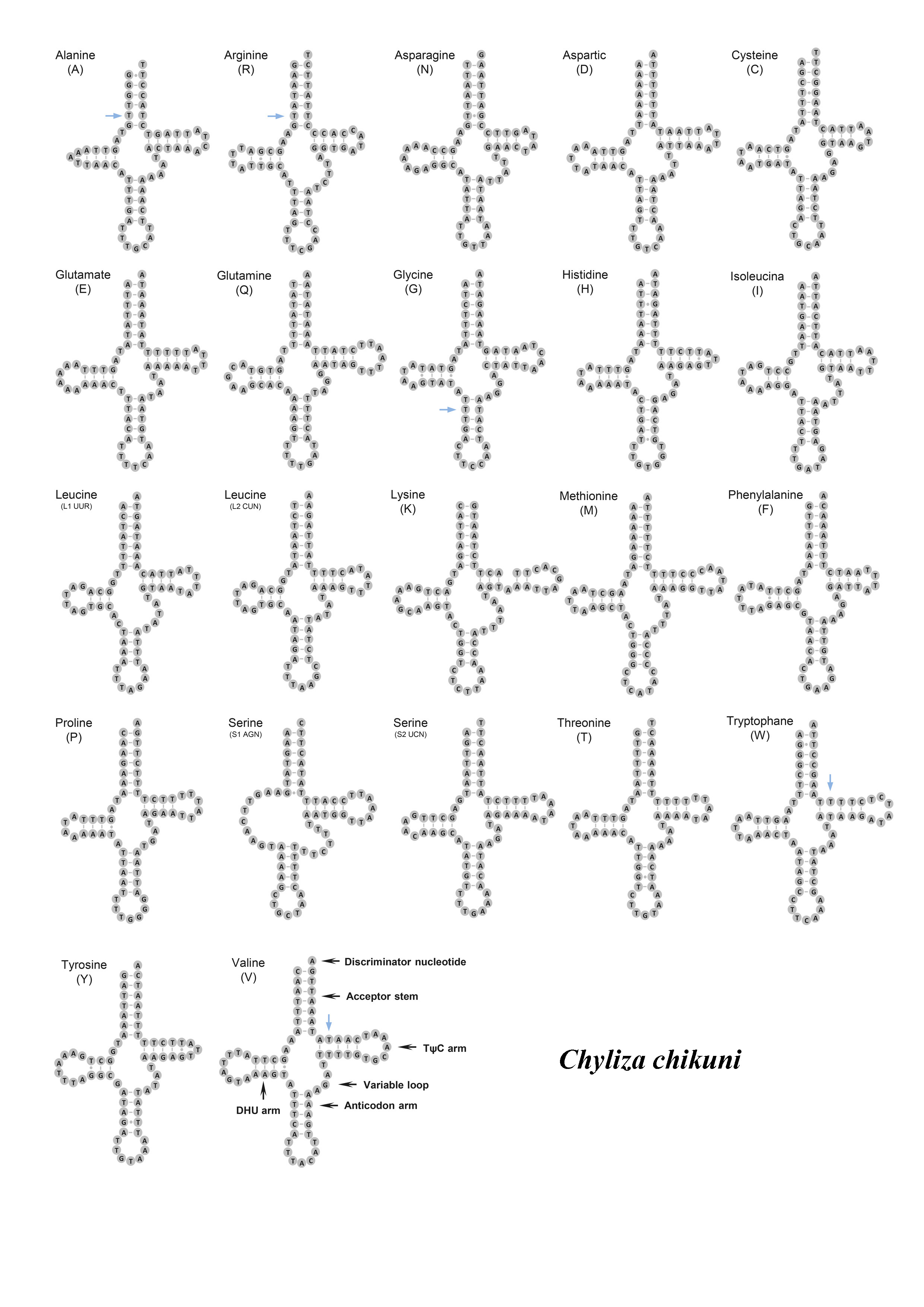

Supplement: Supplementary file 1 [file insects-13-00518-s001.zip › Figure S2-3.jpg]

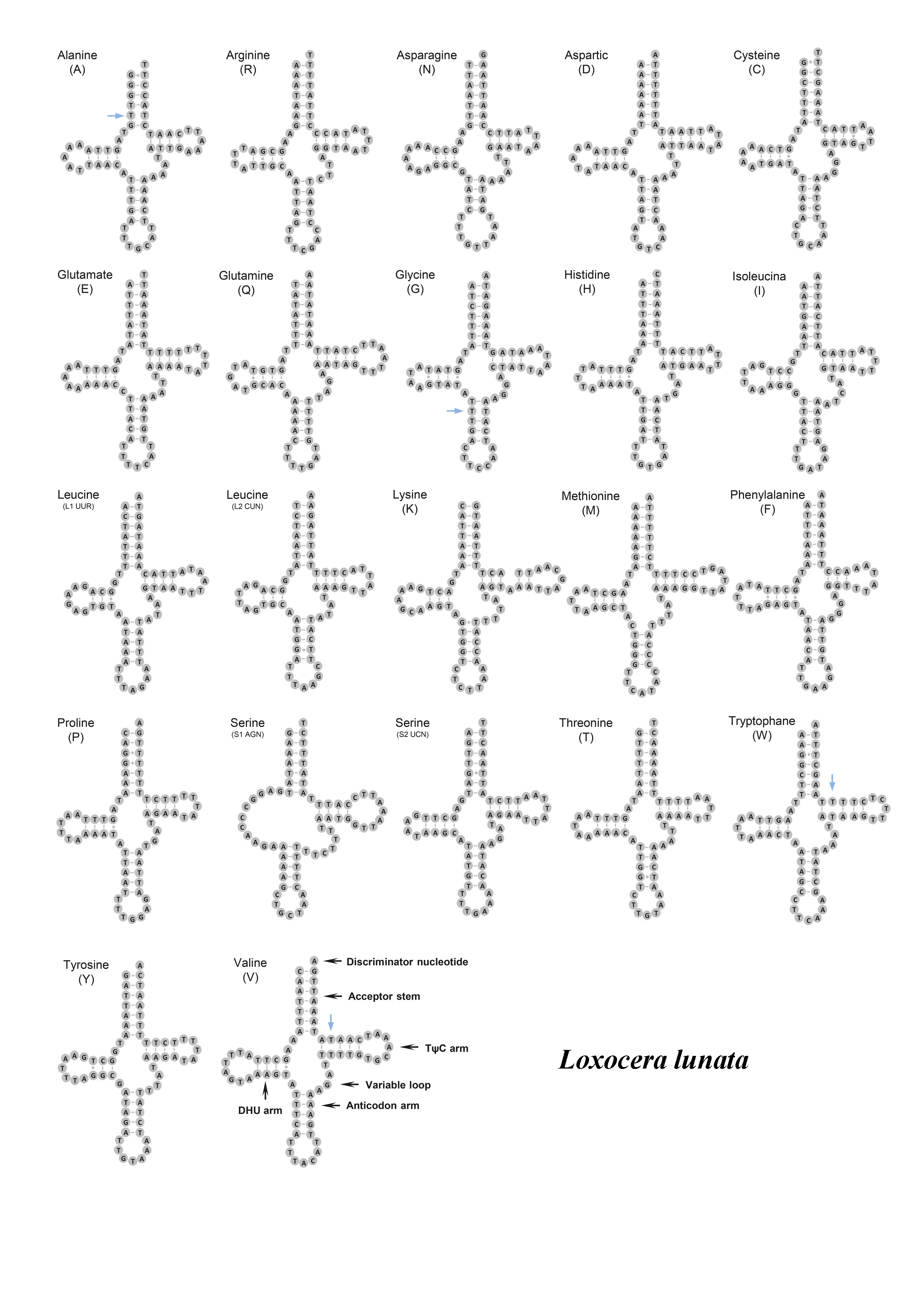

Supplement: Supplementary file 1 [file insects-13-00518-s001.zip › Figure S2-4.jpg]

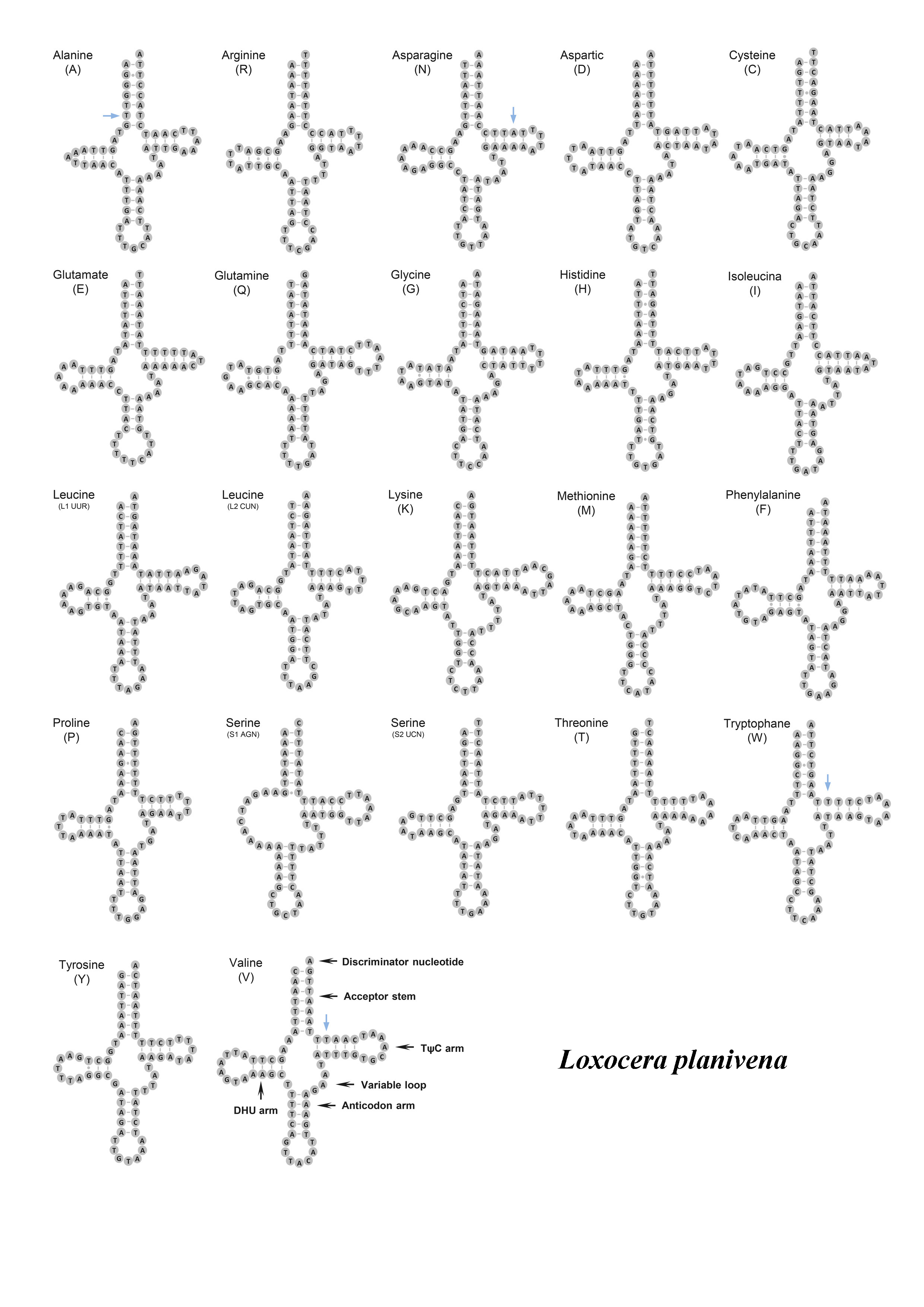

Supplement: Supplementary file 1 [file insects-13-00518-s001.zip › Figure S2-5.jpg]

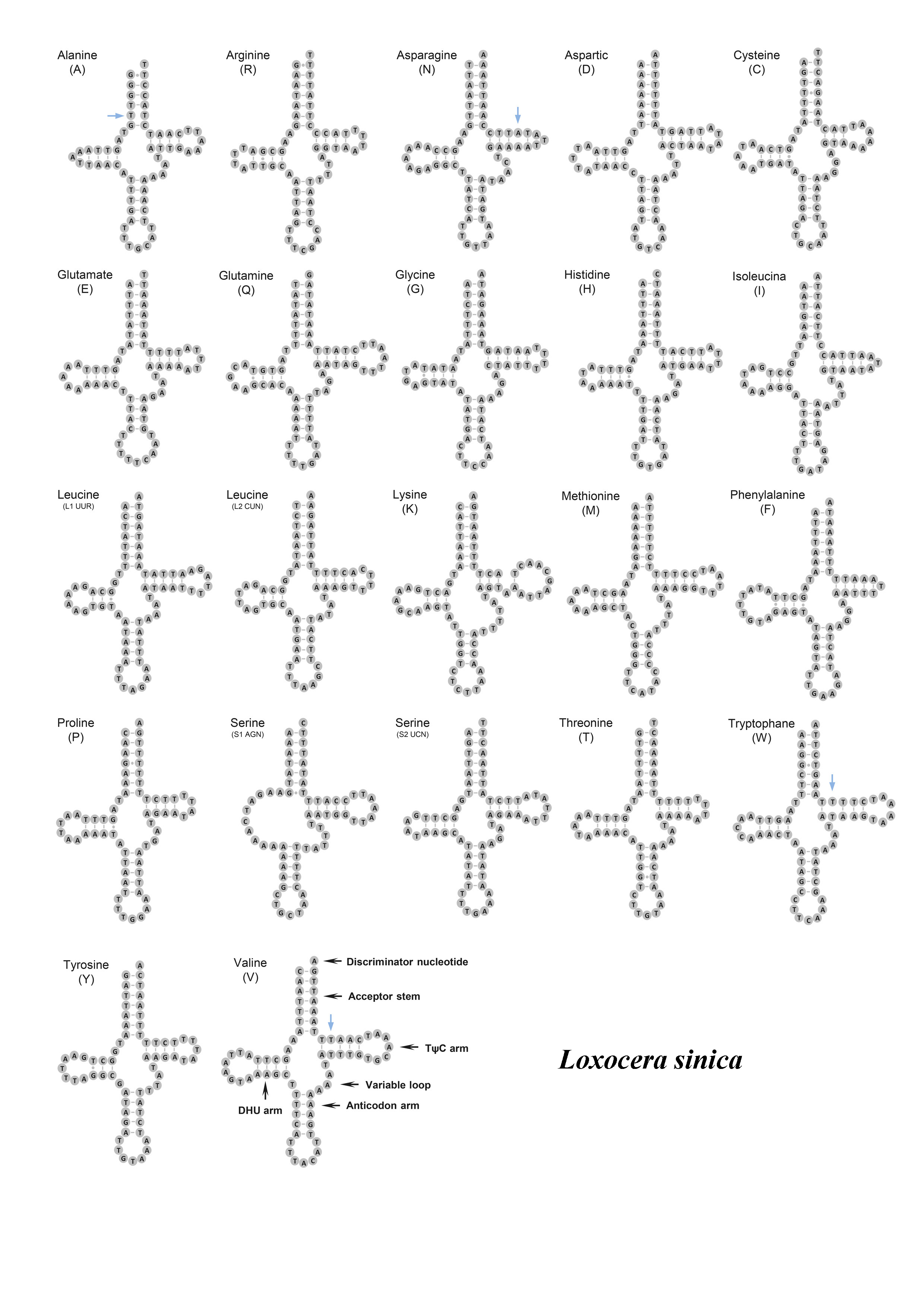

Supplement: Supplementary file 1 [file insects-13-00518-s001.zip › Figure S2-6.jpg]
